# Clinical analysis of infectious mononucleosis complicated with acute acalculous cholecystitis

**DOI:** 10.3389/fped.2024.1339920

**Published:** 2024-03-08

**Authors:** Caijie Gao, Liming Cao, Xiaoli Mei

**Affiliations:** Department of Infectious Disease, Children’s Hospital of Nanjing Medical University, Nanjing, China

**Keywords:** children, infectious mononucleosis, acute acalculous cholecystitis, clinical analysis, Epstein-Barr virus

## Abstract

**Objective:**

This study aimed to investigate specific clinical diagnostic methods for children with infectious mononucleosis (IM) complicated by acute acalculous cholecystitis (AAC).

**Methods:**

We conducted a retrospective analysis of 171 cases of IM diagnosed in the infectious disease ward of Children's Hospital of Nanjing Medical University between January 2020 and December 2020. All IM patients underwent abdominal ultrasound examinations to assess the liver, gallbladder, and spleen. Fourteen patients with symptoms of AAC underwent a follow-up assessment one week later.

**Results:**

The estimated incidence of AAC in hospitalized IM children was 8.2%. Both groups of patients presented with fever, abdominal pain, and eyelid edema upon admission. Characteristic radiological findings of AAC were observed, including gallbladder (GB) distention, increased GB wall thickness and increased common bile duct diameter. Analysis of laboratory results revealed no statistically significant differences in leukocyte, absolute lymphocyte count, CD3+, CD3 + CD4+, CD3+ CD8+, Aspartate Aminotransferase (AST), Alanine Aminotransferase (ALT), or Gamma-Glutamyl Transferase (GGT) levels between the AAC(+) and AAC(−) groups on admission. However, these parameters were not significant risk factors for AAC. After discharge, relevant indicators in non-AAC patients gradually decreased to normal levels, while those in AAC(+) patients did not show a significant decrease.

**Conclusion:**

While cases of IM complicated by AAC are relatively uncommon, the utilization of abdominal ultrasound offers a reliable tool for confirming this diagnosis. Routine abdominal ultrasound examinations are recommended for IM patients to improve early detection and treatment of associated conditions.

## 1 Introduction

Acute acalculous cholecystitis (AAC) frequently presents as a complication of severe infectious mononucleosis, representing a more serious clinical manifestation of the disease. Notably, the clinical presentation of AAC in this context often lacks specific diagnostic indicators (1). Importantly, gallbladder diseases are uncommon in children, and gallbladder inflammation in this age group is particularly rare. However, approximately 30%–50% of childhood cases of gallbladder inflammation manifest as acalculous cholecystitis (1). In most cases, AAC is associated with various infectious diseases, including streptococcal infections, hepatitis A, IM, viral enteritis, leptospirosis, salmonellosis, ascariasis, or giardiasis (2). Additionally, reports have shown its concurrent occurrence with some non-infectious systemic diseases such as Kawasaki disease, trauma-induced rhabdomyolysis, toxic epidermal necrolysis, hemolytic-uremic syndrome, and long-term total parenteral nutrition (3). Certain malignant tumors, particularly hemophagocytic lymphohistiocytosis, and long-term total parenteral nutrition, have also been documented as risk factors for AAC (1). Furthermore, congenital gallbladder deformities, congenital biliary anomalies, and acquired diseases leading to bile stasis have been reported as risk factors for pediatric gallbladder inflammation (4–6).

Although the exact pathogenic mechanism of AAC remains unclear, gallbladder ischemia is generally considered one of its fundamental pathological features, likely resulting from multiple mechanisms (6). The primary blood supply to the gallbladder is the cystic artery, which arises from the narrow terminal branch of the right hepatic artery. This explains the gallbladder's vulnerability to ischemia (4). It is now understood that factors including malnutrition, intravenous opioid use, or gallbladder duct obstruction can lead to gallbladder dysfunction and bile stasis, contributing to ischemia. Additionally, other factors that cause local hypoperfusion, such as cardiovascular surgery, congestive heart failure, shock, and arterial occlusion (e.g., vasculitis), can also result in gallbladder ischemia (5). Sepsis can further contribute to gallbladder wall inflammation by generating inflammatory and vasoactive mediators (5, 7).

While inadequate perfusion and subsequent gallbladder ischemia can explain the pathogenesis of AAC in critically ill and/or shock patients, this mechanism does not apply to non-critically ill children with AAC (8). This latter group commonly presents with severe infections and injuries, often requiring antibiotic therapy and/or surgical intervention. Although AAC can manifest as a complication of primary Epstein-Barr virus (EBV) infection, its clinical presentation is atypical (9). The precise mechanistic underpinnings of EBV-related acute cholecystitis remain elusive, with potential contributions from direct EBV invasion and bile stasis secondary to gallbladder irritation (10). Notably, severe infectious mononucleosis can be complicated by AAC, with gallbladder ultrasonography serving as the most reliable tool for early detection.

To further investigate the relationship between infectious mononucleosis IM and AAC, we conducted a retrospective analysis of clinical data from 171 pediatric IM cases with concurrent AAC. Based on the presence or absence of AAC, we categorized the patients into the AAC group and the non-AAC group. Our study revealed that the proportion of IM patients with AAC is relatively low. In AAC patients, serum T cells and T suppressor/killer cells were found to be highly expressed. However, due to the lack of specificity, ultrasound examination of the gallbladder remains a reliable diagnostic method for confirming AAC.

## 2 Materials and methods

### 2.1 Study design and population

We selected 171 pediatric patients diagnosed with IM admitted to the Department of Infectious Diseases at Children's Hospital of Nanjing Medical University from January 2020 to December 2020. The study design was reviewed and approved by the Medical Ethics Committee of the Affiliated Children's Hospital of Nanjing Medical University (202402010-1).

Inclusion criteria: Children included in the study met all diagnostic criteria for infectious mononucleosis (11), possessed complete clinical data, and had no other concurrent bacterial or viral infections. Both positive EBV antibody and EBV DNA tests were required for inclusion. The diagnosis of AAC was based on the following four criteria (12): gallbladder (GB) distention, GB wall thickness exceeding 3.5 mm, non-shadowing echogenic sludge, and pericholecystic fluid collection.

Exclusion criteria: Children with other viral infections, immune system defects impacting study progress, or underlying conditions affecting absolute lymphocyte count (malnutrition, leukemia, hematopoietic stem cell transplantation, etc.) were excluded.

Treatment: Both groups received the same drug regimen: acyclovir for antiviral treatment and vitamin C for symptom management.

### 2.2 Data collection

Clinical data for all study subjects were collected from the electronic medical record system, including demographics (age, sex), symptoms (fever, eyelid edema, abdominal pain, lymph node enlargement), physical exam findings (liver, spleen), and laboratory results (blood tests for leucocytes, atypical lymphocytes, absolute lymphocyte count, C-reactive protein (CRP), Alanine Aminotransferase (ALT), Aspartate Aminotransferase (AST), glutamyl transpeptidase (GGT) activity, T lymphocytes (CD3+), CD3 + CD4+ T lymphocytes (helper T cells), CD3 + CD8+ T lymphocytes (cytotoxic T cells), EBV antibody levels, EB-DNA levels in serum, and gallbladder B ultrasound results.

### 2.3 Laboratory tests and ultrasound tests

Upon admission, all children underwent abdominal ultrasound using a commercially available high-end Philips iU 22 instrument with 7–12 MHz linear and 4–9 MHz convex probes, and venous blood was drawn for complete blood count (Mindray 7,500 Hempocyte), atypical lymphocytes, Epstein-Barr virus antibodies and DNA (immunoturbidimetry-Roche 502 and PCR fluorescence), CD3+, CD3 + CD4+, CD3 + CD8+ (immunoturbidimetry-Roche 502), and liver enzymes (AST, ALT, GGT) analysis (automatic biochemical analyzer-Roche 702). All children also underwent a transabdominal liver ultrasound in the supine and right anterior oblique positions by a qualified ultrasound imaging physician.

### 2.4 Statistical methods

Graphpad Prism 9.0 was used for graphical representation and statistical analysis. Quantitative data were represented by Median (25%, 75%) or mean ± standard error (sem); count data were represented as cases (%). The normality of quantitative data was checked by Shapiro-Wilk test. Nonparametric test, specifically, the Mann-Whitney for two groups and Kruskal-Wallis tests for three groups, were employed to analyze the differences in laboratory examinations, including leucocytes count, absolute lymphocyte count, CD3+, CD3 + CD4+, CD3 + CD8+, ALT, AST, GGT, and age among confirmed IM patients with AAC. A *P*-value <0.05 was considered statistically significant.

## 3 Results

### 3.1 Basic characteristics of patients

Our study included 171 children aged between 9 months and 15 years (mean age: 49.96 ± 30.40 months, median: 43.0), with male predominance (*n* = 99/171, 57.9%). Fourteen children developed severe AAC symptoms, constituting the AAC(+) group. This group comprised 6 girls and 8 boys, with ages ranging from 2 to 12 years (mean age: 51.64 ± 30.61 months, median: 42.5), and included 13 children younger than 7 years. The remaining 157 children, forming the AAC(−) group, exhibited an onset age predominantly between 0 and 7 years (89.9%). This group included 91 boys and 66 girls, with ages ranging from 2 to 12 years (mean age: 49.82 ± 30.48 months, median: 43.0). Notably, 142 children in the AAC(−) group presented within the 0–7 age range. A detailed breakdown of these demographics is presented in Table 1.

**Table 1:** Basic picture and clinical indications of children with IM (*n*, %).

		ACC−	ACC+	*P*-value
Group *n* (%)		157 (91.8)	14 (8.2)	
Sex (*n*)	Boy	91	8	0.953
	Girl	66	6	
Age (years)	0–7	141	13	0.999
	7–12	16	1	
	Fever (+)	137	13	0.999
Clinical presentation at admission (*n*)	Abdominal pain (+)	6	2	0.131
	Eyelid edema (+)	50	9	0.020*
	Murphy's (+)	0	0	-
	VCA-IgM (+)	145	12	0.321
	VCA-IgG (+)	40	3	
	EBNA-IgG (+)	29	3	
EBV testing (*n*)	EB-DNA (<500)	38	1	0.194
	EB-DNA (>500)	144	13	0.999

EBV, Epstein-Barr virus; EBNA, EBV nuclear antigen; VCA, viral capsid antigens.

* *p* < 0.05 as statistically significant.

### 3.2 Clinical manifestations

Analysis of clinical characteristics revealed that 92.9% of children in the AAC(+) group presented with fever, compared to 87.2% in the AAC(−) group. This difference was not statistically significant (*P* > 0.05). The prevalence of abdominal pain and double eyelid edema was higher in the AAC(+) group than the AAC(−) group, although all children were negative for Murphy's sign. This may be attributed to the younger age of the participants and the limitations of physical examination in this population. Serological testing for EBV antibodies showed positivity in 85.7% of the AAC(+) group and 92.4% of the AAC(−) group (*P* > 0.05). Further EB-DNA testing revealed elevated levels (>500) in 92.9% of AAC(+) children and 72.6% of AAC(−) children. While statistically insignificant (*P* > 0.05), the median EB-DNA titer was higher in the AAC(+) group (20,483 ± 13,667) compared to the AAC(−) group (8,114 ± 4,742). These findings suggest a potential association between higher EB-DNA titers and AAC development. A detailed summary of the clinical characteristics is presented in Table 1.

### 3.3 Ultrasound examination

Ultrasound imaging revealed highly aberrant findings in patients with AAC. Within the AAC(+) group, all participants displayed marked gallbladder enlargement, accompanied by substantial thickening of the gallbladder wall and significant dilation of the common bile duct. Notably, 69% of patients within this group additionally demonstrated gallbladder wall edema while maintaining normal echogenicity within the gallbladder lumen (Table 2).

**Table 2:** Ultrasonography findings in children with AAC (*n* = 14).

		Clinical examinations		Gallbladder		
Gender/age (year)	Symptoms	H (cm)	S (cm)	Size (mm^2^)	Thickness (mm)	Gallbladder wall edema
B/5	Fever, pain, nausea, vomiting, eyelid edema	2.8	2.8	53 × 20	4–5	Yes
G/3	Fever, eyelid edema	2.8	1.2	41 × 18	No	No
G/2	Fever	2.4	1.1	42 × 24	4	Yes
G/6	Fever	2.5	2.5	68 × 26	5	Yes
B/5	Fever, pain, eyelid edema	3.2	5.1	39 × 8	5–6	Yes
G/2	Fever, eyelid edema	3.5	0	50 × 20	4	Yes
G/3	Eyelid edema	2.6	2.9	45 × 17	No	No
B/5	Fever	2.9	2.2	56 × 26	4–5	Yes
B/2	Fever, eyelid edema	0	1.4	37 × 16	4–5	Yes
B/2	Fever, eyelid edema	1.7	0	49 × 18	4.5	Yes
B/4	Fever, eyelid edema	3	2.5	61 × 20	6–7	Yes
G/11	Fever	3.8	3.1	83 × 29	No	No
B/3	Fever, eyelid edema	3	2	56 × 20	4–5	Yes
B/2	Fever	2.5	3.1	40 × 20	5–6	Yes

H, hepatomegaly (cm below ribs); S, splenomegaly (cm below ribs); B, boy; G, girl.

### 3.4 Laboratory examinations

**Figure 1:**
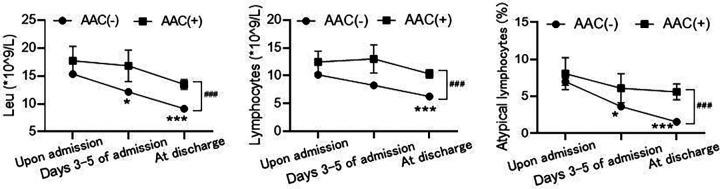
Comparison of routine blood test results between the AAC(+) and AAC(−) groups. The values of leukocytes (Leu, left), absolute lymphocytes (LYMPH, middle), and atypical lymphocytes (right) between the two groups at different time points: upon admission, days 3−5 of admission and at discharge. *P < 0.05, ***P < 0.001, compared to the level of upon admission in AAC(−) group by Kruskal-Wallis tests; ^###^*P* < 0.001, the difference between AAC(+) and AAC(−) groups at discharge by Mann-Whitney test.

Analysis of peripheral blood leukocyte counts revealed no statistically significant differences in leukocytes, absolute lymphocytes, and atypical lymphocytes between the AAC(+) and AAC(−) groups upon admission across all age groups. However, the AAC(−) group showed a time-dependent decrease in these values at 3–5 days and upon discharge, while the AAC(+) group did not. Levels in the AAC(+) group remained consistent with admission levels at 3–5 days and were significantly higher upon discharge than in the AAC(−) group, suggesting a potential persistent inflammatory response in the AAC(+) group (Table 3 and Figure 1).

**Table 3:** The results of laboratory analysis in children (mean in the group).

	AAC(−)	AAC(+)	*P*-value
Leu (×10^9^/L)			
Upon admission	15.4	17.8	0.45
Days 3–5 of admission	12.2	16.8	0.04*
At discharge	9.1	13.5	0.001**
Lymphocytes count (×10^9^/L)			
Upon admission	10.2	12.5	0.26
Days 3–5 of admission	8.3	13	0.01*
At discharge	6.3	10.3	0.001**
Atypical lymphocytes (%)			
Upon admission	7	8.1	0.78
Days 3–5 of admission	3.6	6.1	0.24
At discharge	1.6	5.6	0.001**
CRP (>8 mg/dl)			
Upon admission (%)	27	14	
Days 3–5 of admission (%)	13	0	
At discharge (%)	0	0	
GGT (U/l)			
Upon admission	58	96.8	0.12
At discharge	37.7	86.8	0.008**
ALT (U/l)			
Upon admission	160.4	170.2	0.67
At discharge	67.9	70.9	0.56
AST (U/l)			
Upon admission	98.2	148.8	0.08
At discharge	51.3	54.3	0.56
CD3 + (count/ul)	11,292	7,180	0.006**
CD3 + CD4 + (count/ul)	1,503	1,380	0.38
CD3 + CD8 + (count/ul)	8,704	5,020	0.003**

Leu, leucocytes; CRP, C-reactive protein; ALT, alanine aminotransferase; AST, aspartate aminotransferase; GGT, gamma glutamyl transpeptidase; CD3+, T lymphocytes; CD3 + CD4+, T assisted/induced lymphocytes; CD3 + CD8+, T inhibits/kills lymphocytes.

* *p* < 0.05.

** *p* < 0.01 as statistically significant.

**Figure 2:**
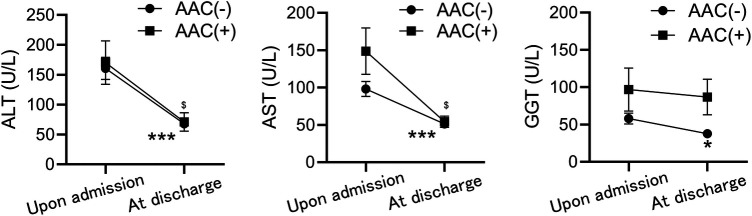
Comparison of liver function test results between the AAC(+) and AAC(−) groups. The values of ALT (left), AST (middle), and GGT (right) between the two groups upon admission, and at discharge. *P < 0.05, ***P < 0.001, ^$^*P* < 0.05, compared to the level of upon admission in AAC(−) and AAC(+), respectively by Mann-Whitney test.

Liver function tests indicated no significant differences in ALT, AST, and GGT levels between the two groups upon admission; however, these levels were elevated in both groups. Upon discharge, significant decreases were observed, suggesting gradual recovery of liver function. Nonetheless, AST, ALT, and GGT levels remained consistently higher in the AAC(+) group throughout hospitalization (Figure 2).

**Figure 3:**
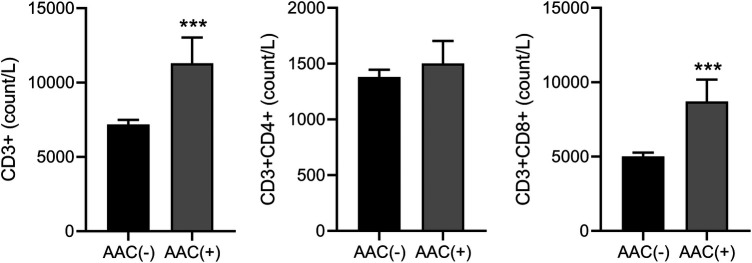
Comparison of immunocell analysis results between the AAC(+) and AAC(−) groups. The value difference of CD3+ (left), CD3 + CD4+ (middle), and CD3 + CD8+ (right) between the two groups. ***P < 0.001, compared to the level in AAC(−) by Mann-Whitney test.

Immunocell analysis showed significantly higher CD3+, CD3 + CD4+, and CD3 + CD8 + levels in the AAC(+) group compared to the AAC(−) group. However, there was no significant difference in the rate of change of these values between the two groups (Figure 3).

In our cases, CRP levels exhibited limited ability to differentiate between AAC(−) and AAC(+) patients. Upon admission, 86% of patients in the AAC(+) group had CRP levels below 8 compared to 73% in the AAC(−) group. Notably, all patients had CRP levels below 8 before discharge. This suggests a possible association between AAC caused by EBV infection and the immune inflammatory response triggered by the virus.

## 4 Discussion

Our findings have demonstrated that all AAC patients in our study display evidence of EBV infection. However, the specific mechanism underlying viral infection-associated AAC in otherwise healthy children remains unclear. While various viruses, such as cytomegalovirus, hepatitis A virus, and dengue virus, have been linked to AAC, each likely follows distinct pathogenic pathways (13). Our study specifically isolated the EBV as a potential risk factor, excluding the aforementioned viral agents. By analyzing the clinical presentation of combined EBV infection and cholecystitis, we identified a potential incidence of approximately 8.2% for AAC among hospitalized children experiencing severe IM symptoms. Notably, we were unable to identify any previous studies in the analyzed literature that assessed this specific risk factor. Additionally, the increasing number of reported cases of EBV-related AAC suggests that the true prevalence might be currently underestimated (14). This potential underestimation may be further attributed to the fact that our study only included hospitalized children, lacking data on individuals receiving treatment at home. Furthermore, the limited sample size and heterogeneity within our AAC group emphasize the need for future studies with larger and more homogeneous cohorts for robust statistical analysis.

Beyond EBV, bacterial infection remains a potential contributing factor to AAC. Reported etiological agents in systemically infected patients with AAC include bacterial enteritis, typhoid fever, non-infectious salmonellosis, pyogenic streptococcus, Vibrio parahaemolyticus, and pneumococcus, potentially leading to bacterial colonization of the gallbladder (13). In the present study, persistent elevations in leukocyte and atypical lymphocyte counts among the AAC group during hospitalization, without the observed time-dependent decrease seen in the non-AAC group, suggest the possibility of ongoing infection. This finding warrants further investigation to fully preclude the role of direct bacterial involvement in AAC pathogenesis. Previously established literature demonstrates a correlation between peripheral blood CRP concentration and the severity of acute cholecystitis, with some studies suggesting its utility as a reliable predictor of inflammatory burden (15, 16). While this association is more firmly established in adult populations, further validation is necessary to confirm its applicability to pediatric patients. Notably, none of the AAC children in our study received antibiotic treatment, and all exhibited normal gallbladder ultrasound findings in outpatient follow-up. Therefore, the potential benefits of early antibiotic intervention in IM-associated AAC, specifically regarding reduction in disease duration, require further verification.

During primary EBV infection, cellular immunity mediated by CD4 + and CD8+ T lymphocytes plays a complex role, potentially controlling chronic infection while also exacerbating the severity of IM symptoms (17). Therefore, elucidating the dynamics of immune cell changes during EBV-associated IM and AAC is crucial for informed clinical decision-making. This study's findings demonstrate significant alterations in the levels of CD3+, CD3 + CD4+, and CD3 + CD8 + immune cells within the AAC group. This observation suggests that AAC may further induce bodily immune activation, necessitating heightened clinical vigilance to prevent opportunistic infections arising from potential immune system compromise. Moreover, the integrated application of ultrasound criteria and elevated blood markers offers a valuable tool for early AAC detection.

Our analysis revealed significantly elevated mean levels of AST, ALT, and GGT in AAC(+) children. Notably, these indicators exhibited a gradual decline over the course of hospitalization in this group. While AAC is associated with acute hepatitis, the distinct trajectory of these markers during hospitalization suggests that the hepatitis is not a direct consequence of either the inflammation itself or intrahepatic bile cholestasis (18).

The diagnosis of acute acalculous cholecystitis in children is typically based upon a multi-pronged approach, encompassing clinical symptomatology, laboratory analysis, and abdominal ultrasound examinations. Children experiencing EBV-related AAC commonly exhibit right upper quadrant abdominal pain and fever, with occasional nausea and vomiting. These presentations align with observations documented in the literature (10, 14). However, due to the unique developmental characteristics and limited language expression of pediatric patients, atypical clinical presentations can hinder early detection of AAC, necessitating reliance on auxiliary examinations. While the clinical manifestations of AAC lack specificity and may mimic gallstone disease (19), considering the patient's age can be valuable for ruling out certain diagnoses, such as cholelithiasis. In conclusion, during the primary EBV infection period, AAC represents a relatively common but potentially underdiagnosed pathological condition, especially in pediatric populations. Its atypical clinical presentation and absence of definitive laboratory markers necessitate reliance on ultrasound examinations for auxiliary diagnosis, which highlights the potential for misdiagnosis.

## 5 Conclusion

From the retrospective analysis, we found AAC which we previously neglected is uncommon in children with IM associated with EBV, there have showed some laboratory difference between them, such as the level of leukocytes, absolute lymphocytes, CD3+. But there were no specific and reliable biomarkers that can be identified. So further research should be conducted to be responsible. Our analysis revealed significantly elevated levels of leukocytes, absolute lymphocytes, CD3+, CD3 + CD4+, CD3 + CD8+, AST, ALT, and GGT in the AAC(+) group compared to the control group. However, the limitations of this study's sample size necessitate further investigation to confirm the proposed risk factors. Current routine laboratory tests remain insufficient for early detection of AAC in children. Therefore, we recommend incorporating routine gallbladder ultrasound examinations for children diagnosed with infectious mononucleosis associated with EBV to enable the timely implementation of preventive measures and mitigate the risk of AAC development.

## Data Availability

The original contributions presented in the study are included in the article/**Supplementary Material**, further inquiries can be directed to the corresponding authors.
