# Supplementary material for: Clinical analysis of infectious mononucleosis complicated with acute acalculous cholecystitis
Source: Front Pediatr. 2024 Mar 8;12:1339920. doi: 10.3389/fped.2024.1339920 (PMC10957744; doi:10.3389/fped.2024.1339920)
Supplement: Supplementary file 1 [file Table1.pdf]

Supplemental Table 1. The values of leukocytes (Leu) between AAC(+) and AAC(-) groups at three time points: upon admission, days 3-5 of admission and at discharge.

Percentile	Leu(×10^9/L)					
	AAC(+)			AAC(-)		
	Upon admission	Days 3-5 of admission	At discharge	Upon admission	Days 3-5 of admission	At discharge
25%	10.7	12.0	11.6	11.8	9.6	7.0
50%	16.7	16.0	12.9	14.8	11.3	8.8
75%	23.3	19.7	15.9	18.7	14.5	10.5
Mean	17.8	16.8	13.5	15.4	12.2	9.1
sem	2.6	2.8	0.8	0.4	0.4	0.2

Supplemental Table 2. The values of absolute lymphocytes between AAC(+) and AAC(-) groups at three time points: upon admission, days 3-5 of admission and at discharge.

Percentile	Absolute lymphocytes (×10^9/L)					
	AAC(+)			AAC(-)		
	Upon admission	Days 3-5 of admission	At discharge	Upon admission	Days 3-5 of admission	At discharge
25%	7.4	9.0	8.4	7.3	6.5	4.6
50%	11.7	11.7	9.4	9.5	7.7	5.7
75%	16.0	15.3	12.3	12.6	10.2	7.8
Mean	12.5	13.0	10.3	10.2	8.3	6.3
sem	1.9	2.5	0.7	0.3	0.3	0.2

Supplemental Table 3. The values of atypical lymphocytes between AAC(+) and AAC(-) groups at three time points: upon admission, days 3-5 of admission and at discharge.

Percentile	atypical lymphocytes (%)					
	AAC(+)			AAC(-)		
	Upon admission	Days 3-5 of admission	At discharge	Upon admission	Days 3-5 of admission	At discharge
25%	2.8	0.0	2.5	3.0	2.0	0.0
50%	6.5	5.0	5.0	5.0	3.0	0.0
75%	10.5	10.0	8.5	10.0	5.0	3.0
Mean	8.1	6.1	5.6	7.0	3.6	1.6
sem	2.2	1.9	1.1	0.5	0.4	0.2

Supplemental Table 4. The values of ALT, AST, and GGT between AAC(+) and AAC(-) groups upon admission, and at discharge.

Percentile	ALT (U/L)				AST				GGT			
	AAC(+)		AAC(-)		AAC(+)		AAC(-)		AAC(+)		AAC(-)	
	Upon admission	At discharge	Upon admission	At discharge	Upon admission	At discharge	Upon admission	At discharge	Upon admission	At discharge	Upon admission	At discharge
25%	41.0	29.3	61.8	31.0	58.0	34.8	44.8	33.0	19.75	35.5	14.0	15.0
50%	181.0	57.0	110.5	47.5	130.0	41.0	74.0	43.0	71.5	60.5	30.0	25.0
75%	284.0	102.3	190.5	79.0	199.3	75.0	115.3	55.3	115.3	91.5	68.0	41.8
Mean	170.2	70.9	160.4	67.9	148.8	54.3	98.2	51.3	96.8	86.8	58.0	37.7
sem	36.4	15.3	18.6	7.0	31.2	7.5	10.2	3.7	28.8	24.0	7.3	4.2

Supplemental Table 5. The value difference of CD3+, CD3+CD4+, and CD3+CD8+ between AAC(+) and AAC(-) groups.

Percentile	CD3+(count/ul)		CD3+CD4+(count/ul)		CD3+CD8+(count/ul)	
	AAC(+)	AAC(-)	AAC(+)	AAC(-)	AAC(+)	AAC(-)
25%	4543	6800	785	960	2808	5299
50%	6718	10776	1118	1362	4458	8252
75%	9225	13379	1810	1834	6885	10739
Mean	7180	11292	1380	1503	5020	8704
sem	312	1745	66	202	251	1453
